# A Comprehensive Investigation on Multi-User Interference Effects in Vehicular Visible Light Communications

**DOI:** 10.3390/s23052553

**Published:** 2023-02-24

**Authors:** Emmanuel Plascencia, Hongyu Guan, Luc Chassagne, Olivier Barrois, Oyunchimeg Shagdar, Alin-Mihai Căilean

**Affiliations:** 1 Oledcomm Company, 78140 Vélizy, France; 2 Systems Engineering Laboratory of Versailles, University of Versailles Saint-Quentin-en-Yvelines, University of Paris-Saclay, 78140 Vélizy, France; 3 Renault Group, 78084 Guyancourt, France; 4 Department of Computers, Electronics and Automation, Stefan Cel Mare University of Suceava, 720229 Suceava, Romania; 5 Integrated Center for Research, Development and Innovation in Advanced Materials, Nanotechnologies and Distributed Systems for Fabrication and Control, Stefan Cel Mare University of Suceava, 720229 Suceava, Romania

**Keywords:** inter-vehicle communications, multi-user interference, optical interference, V2V, vehicle safety, VLC, visible light communication

## Abstract

Vehicular visible light communications (VLC) are considered a suitable technology for vehicular platooning applications. Nevertheless, this domain imposes strict performance requirements. Although numerous works have shown that VLC technology is compatible with platooning applications, existing studies are mainly focused on the physical layer performances, mostly ignoring the disruptive effects generated by neighboring vehicular VLC links. Nevertheless, the 5.9 GHz Dedicated Short Range Communications (DSRC) experience has shown that mutual interference can significantly affect the packed delivery ratio, pointing out that these effects should be analyzed for vehicular VLC networks as well. In this context, this article provides a comprehensive investigation focused on the effects of mutual interference generated by neighboring vehicle-to-vehicle (V2V) VLC links. Therefore, this work provides an intensive analytical investigation based on simulation and also on experimental results that demonstrate that although ignored, the influence of mutual interference is highly disruptive in vehicular VLC applications. Hence, it has been shown that without preventive measures, the Packet Delivery Ratio (PDR) can decrease below the imposed 90% limit for almost the entire service area. The results have also shown that although less aggressive, multi-user interference affects V2V links even in short-distance conditions. Therefore, this article has the merit of emphasizing a new challenge for vehicular VLC links and points out the importance of multiple-access techniques integration.

## 1. Introduction

**Figure 1:**
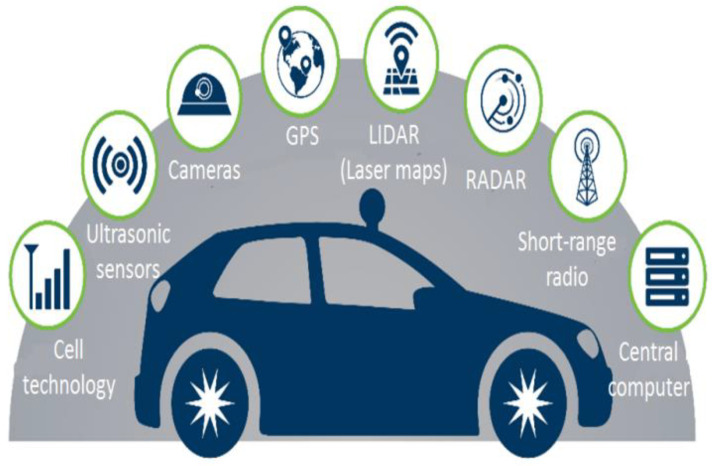
Illustration of the complexity of modern vehicles including numerous types of sensors: ultrasonic sensors, camera systems, GPS, LIDAR, radar, a central computer that process the data and communication systems that enable information exchange.

Nowadays, modern vehicles have advanced from metal shells on wheels to highly complex sensing and computation-capable equipment, as shown in Figure 1. Therefore, hundreds of embedded sensors and the associated processing tools are now standard on high-end version vehicles, enabling them to perceive their surroundings and interact with the environment in semi-autonomous, and eventually in completely autonomous ways. The road infrastructure has also developed, including adaptive traffic signals and communication-capable pay tolls installed on roadways. Next, it is expected that by adopting the concept of communication and enabling information exchange between vehicles and infrastructure, the evolution of road transportation systems will be taken to the next level, as these technologies have the potential to address more than 81% of the traffic accidents, providing significantly improved traffic efficiency [1,2,3,4,5].

In terms of wireless vehicular communications enabling technologies, 5.9 GHz Dedicated Short Range Communications (DSRC) regulated by the IEEE 802.11p standard represent the most mature solution [4,5]. Nevertheless, there are works that indicate that in high-vehicle-density applications (i.e., crowded roads, multiple-lane highways, crowded intersections), the high number of nodes (i.e., communicating vehicles), the high message-generation rate (i.e., 10 Hz), and the wide coverage area (i.e., up to 1000 m, with omnidirectional antennas) can generate mutual interference that effect the reliability of DSRC links in terms of Packet Delivery Ratio (PDR) and latencies (i.e., communication-based vehicle applications require latencies that can be below 20 ms) [6,7,8,9].

In the above-mentioned context, Visible Light Communications (VLC) represent a possible solution that could be used as a complementary technology for 5.9 GHz DSRC systems, enabling improved overall communications performance and better resilience to interference and jamming attacks [7,8,9]. Although the use of the VLC technology in vehicular applications is very challenging [10,11] due to an extremely dynamic, very unfriendly, and highly unpredictable communications channel [10,11,12,13], existing vehicular VLC prototypes have made significant progress, being almost ready for market deployment. Thus, the resilience to optical noise of these systems has greatly improved, with existing prototypes being able to support data exchange in strong sunlight conditions [14,15]. Moreover, the communication range has increased up to almost 200 m [16,17] whereas latencies below 20 ms have been demonstrated as well [15,18]. Additionally, vehicular VLC systems can work in light-dimming conditions [19], while also being able to provide inter-vehicle distance measurements and vehicle positioning as an alternative to the GPS solution [20,21,22]. If, in the initial development stage, vehicular VLC receivers with narrow Field-of-View (FoV) were developed to have better resilience to optical noise, existing solutions now have ±20–±50° reception angles to enable communication not only in perfect Vehicle-to-Vehicle (V2V) alignment, but also in cases where vehicles are located on different lanes of the road [22]. Consequently, as the coverage of vehicular VLC receivers is increasing, the hidden node problem arises in VLC networks as well [23]. Therefore, as experimentally demonstrated in [23], without any countermeasures, hidden nodes significantly affect the Packet Delivery Ratio (PDR) and the overall latencies of VLC networks.

Platooning applications are a special use case for VLC technology. In such cases, VLC is favored by a relatively short inter-vehicle distance which usually ensures a higher Signal to Noise Ratio (SNR). According to [1,2,24], an important aspect that should be supported in platooning applications is a PDR higher than 90%. Existing works have confirmed the fact that the VLC technology can support platooning requirements in numerous scenarios [25,26]. Nevertheless, different from the case of the 5.9 GHz DSRC technology, the effect of mutual user interference generated by neighboring vehicular VLC links has not been investigated in these studies.

In light of the upper-mentioned context, this article approaches the issues associated with Multi-Users Interference (MUI) generated by neighboring inter-vehicle VLC links. As far as we know, this is one of the first works that provides a comprehensive analysis of these interferences based on inter-vehicle usage scenario evaluation and also based on experimental determinations with vehicular VLC prototypes. It is important to re-emphasize that the problems associated with mutual interference have been intensively studied in the case of 5.9 GHz DSRC systems [6,7,8,9], and that multiple access solutions have been developed based on this experience. Consequently, in the context in which previous experience has shown that mutual interference can affect the functionality of other communication technologies, such studies are unquestionably required for vehicular VLC links as well.

On the other hand, the development of vehicular VLC prototypes has been mainly focused on PHY layer development, whereas MAC development has been rather neglected, being addressed in relatively few works [27,28,29]. Nevertheless, the results presented in this work emphasize the necessity of the MAC layer development in parallel to the PHY layer development, as also suggested in [28,30,31,32]. The issues associated with multi-users interference have been partially addressed in [31]. Nevertheless, different from [31], this new article provides a more detailed analysis, a more accurate modeling, envisions an extended area, analyses the case when the interference is generated from more than one interfering vehicle, and for different V2V configurations, while most importantly, it also provides experimental results to sustain the simulation ones. Thus, this new article provides one of the most comprehensive investigations focused on multi-users interference in vehicular VLC applications. Another important aspect that needs to be emphasized right from the start is that due to the complexity of the MUI issues, this article cannot address all issues associated with MUI in vehicular VLC applications. Consequently, this article is strictly focused on i. describing and modeling the MUI effect, ii. simulate its effect on VVLC links, and iii. experimentally measure and confirm its effects. Therefore, the aspect related to MUI mitigations will be addressed in [33].

The rest of this work is organized as follows. Section 2 approaches the issues related to mutual interference in vehicular VLC applications in an analytical manner, providing simulation results that confirm the disruptive effect of these conditions. Section 3 details some of the aspects related to the practical implementation of a VLC prototype that is used for the experimental investigation of mutual interference effects, and also delivers the experimental results on this topic, together with a discussion of these results. Next, Section 4 ends this article by providing the conclusions of this work and some future vehicular VLC development strategies.

## 2. Analytical Evaluation of Multi-User Interference in Vehicular Visible Light Communications Applications

Before proceeding forward, we should remember the basic structure of a VLC system. As presented in [10,11], a VLC system consists in a VLC transmitter and a VLC receiver separated by the free space optical communication channel [12,13,14,15]. The VLC transmitter is a dual-purpose device that modulates the data to send onto the optical power of a lighting device. On the other hand, the VLC receiver is based on a photosensitive device that extracts the data from the modulated light beam. On these grounds, the use of the VLC technology in vehicular VLC applications is very challenging due to the specificity of the outdoor VLC channel [10,11,12,34]. It should be remembered that the vehicular VLC channel is characterized as highly dynamic due to the mobility of the vehicles, very unpredictable due to mobility and due to the impact of weather phenomena, and extremely noisy due to the presence of numerous other sources of light that reach to the photosensitive device.

Considering this, the following section aims to provide an analytical determination of the zone where interference from neighboring V2V VLC links may affect communication performances, together with an assessment of the severity of these effects on the PDR. This model is based on the classical VLC channel that has been initially defined in [34], and which is used in most of the VLC modeling works. As this model has been widely debated in the VLC literature, it will not be reproduced again entirely, but we will only make mandatory clarifications where these are imposed.

### 2.1. Defining the Packet Delivery Requirements

In the case of the standard Intensity Modulation/Direct Detection (IM/DD) VLC method, Equation (1) defines the optical power received by the VLC receiver *P_r_*(*ϕ,ψ*) with respect to the irradiance *ϕ* and incidence *ψ* angles.

(1) $$P_{r}\left(\phi ,\psi \right)=\left\{\begin{array}{c} \frac{A_{r}\left(m_{i}+1\right)}{2\pi d^{2}}P_{t}{cos}^{m_{i}}\left(\phi \right)T_{s}\left(\psi \right)g\left(\psi \right)\cos\left(\psi \right),0\leq \psi \leq \psi_{c1},\\ 0,\;\;\;\;\;\;\;\;\;\;\;\;\;elsewhere \end{array}\right.$$

In Equation (1), *A_r_* is the photodetector active area, *m_i_* is the Lambert coefficient given by the VLC transmitter semi-angle, *d* is the VLC distance, *P_t_* is the transmission power, *T_s_*(*ψ*) is the optical band-pass filter, and *g*(*ψ*) is the gain of the optical concentrator, with additional details on these parameters being provided in [34].

Similar to the IEEE 802.15.7 PHY I 100 kb/s case [35], for simplicity reasons and also for an analysis of the real impact at the PHY layer level, Forward Error Correcting (FEC) codes are not being considered. Additionally, multipath fading is ignored as these effects are limited in vehicular VLC applications [10,11,12,36]. In these conditions, the ability of the receiver to decode the received signal or to determine the PDR correctly is influenced by the Bit-Error Ratio (BER) and by the packet size (*L_b_* expressed in bits) in accordance with Equation (2) [37], whereas the relationship between BER and Signal to Interference Noise Ratio (SINR) for On-Off Keying (OOK) modulation is given by Equation (3) [38].

(2) $$PDR={\left(1-BER\right)}^{L_{b}},$$

(3) $$BER={Q(\sqrt{SINR}=Q\left(\sqrt{\frac{P_{r}}{MUI+\sigma_{tot}}}\right),}^{}$$

In Equation (3), the *Q*-function is frequently exploited to determine the area under the tail of a Gaussian probability distribution function which is defined in Equation (4), *P_r_* is the optical power received by the VLC receiver, *σ_tot_* represents the total noise power (i.e., shot and thermal noise) as is well established in the existing literature, and *MUI* is the overall multi-user interference power.

(4) Q(x)=∫z∞12πe−y22dy,

(5) $$\frac{P_{r}}{MUI+\sigma_{tot}}\geq {SINR}_{th}$$

**Figure 2:**
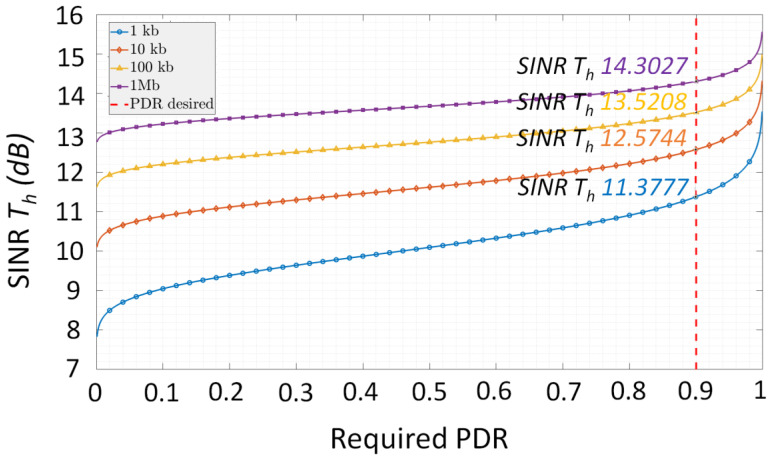
Illustration of the dependency between the required Signal to Interference Noise Ratio threshold with respect to the imposed Packet Delivery Ratio for different packet sizes.

Consequently, it can be deducted from Equation (5) that the received *SINR* must be greater than a specified *SINR* threshold (*SINR_th_*) in order to receive data accurately, whereas the *SINR* threshold can be determined as a function of the imposed communication quality (i.e., the PDR requirement). Since PDR = 1−PER, the *SINR_th_* for a binary modulation system can be deducted based on Equations (2) and (3), leading to Equation (6).

(6) $${SINR}_{th}={\left(Q^{-1}\left(1-\sqrt[{L_{b}}]{{PDR}_{req}}\right)\right)}^{2}$$

where *Q*^−1^ is the inverse *Q* function. Therefore, based on Equation (6), the relationship between *SINR_th_* and PDR can be illustrated. Figure 2 illustrates this dependency for several packet sizes. As one can see, the SINR threshold increases considerably as the PDR threshold increases, reaching 11.37, 12.57, 13.52, and 14.30 dB for a PDR requirement of 90% for a 1, 10, 100 kb and 1 Mb of payload, respectively. Therefore, these results emphasize the high impact of SINR on the PDR. As MUI definitely impacts the SINR, one can connect the MUI with the overall PDR performances.

### 2.2. Performance Modeling in One-User Interference Conditions

**Figure 3:**
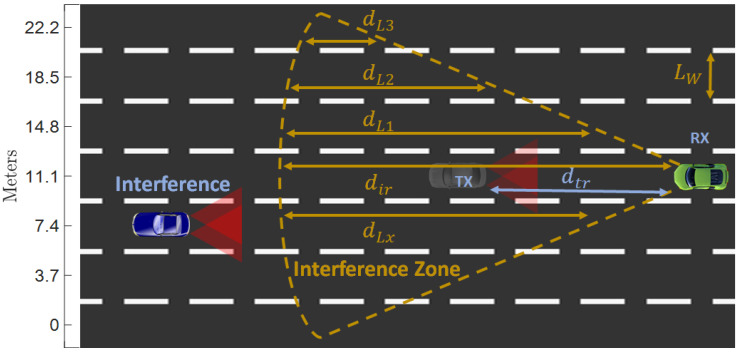
Illustration of the multi-user interference area for a given pair of V2V VLC transmitters (Tx) − receiver (Rx).

After the required *SINR* threshold has been determined, the next step of the analytical determination of the interference effect focuses on establishing the geographical area from which generated interference could affect V2V VLC links. The envisioned communication scenario is illustrated in Figure 3. Although the real-life traffic scenario assumes bidirectional communications involving a VLC tail-lights transmitter and VLC headlight transmitter, these simulations will analyze the most unfavorable situation, which is the case when the tail-light VLC transmitter is used. Here, *d_tr_* is defined as the distance between the intended transmitter (*T_x_*) and the intended receiver (*R_x_*), *d_Lx_* denotes the longitudinal distance within the MUI area for each lane, whereas *L_W_* defines the width of the lanes. For this purpose, the distance between an interfering node and the intended receiver (*d_ir_*) that respects the conditions defined in Equation (7) should be determined.

(7) $$P_{i}\left(d_{ir}\right)\geq \frac{P_{r}\left(d_{tr}\right)}{{SINR}_{th}}-\sigma_{tot}$$

where *P_i_* represents the interference power or the power received from the interfering node. Because VLC is a directional technology, the transmitter and the interfering node must be within the receiving vehicle’s (*R_x_*) FOV. As this article is focused on the effect of neighboring VLC transmitters, the effect of *σ_tot_* from Equation (7) will be ignored, in order to emphasize the effect of MUI. In these conditions, the setup is the equivalent of a V2V link in night conditions. Therefore, as Equation (1) expresses *P_r_* and *P_i_* using *d_tr_* and *d_ir_*, respectively, the highest *d_ir_* that respects the conditions imposed by Equation (7) is given by Equation (8).

(8) $$d_{ir}=d_{tr}\sqrt{{SINR}_{th}}$$

As the interfering nodes and also the intended transmitter must be located within the receiver FOV, the MUI zone for a certain pair of intended *T_x_* and *R_x_* can be defined as the circular segment with a radius *d_ir_* and center angle 2*ψ*, as illustrated in Figure 3. Therefore, this study will provide an estimation of the MUI zone using MATLAB simulations that consider the parameters summarized in Table 1.

**Figure 4:**
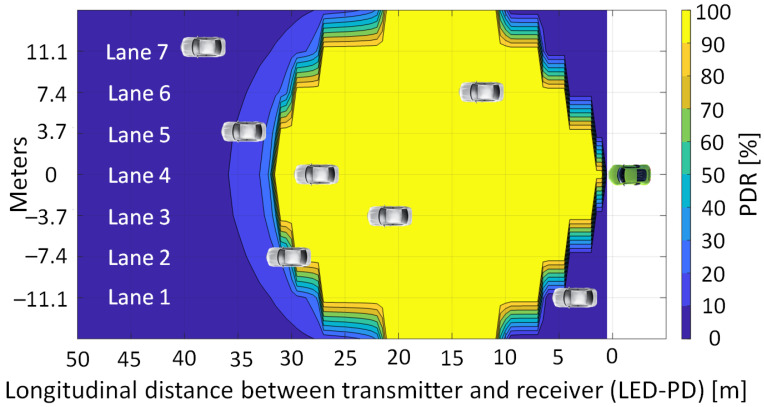
Geographical distribution of the packet delivery ratio for a V2V VLC link when transmitter (white vehicle) and receiver (green vehicle) are on a seven-lane road segment and no interference sources are within the area.

Next, in order to have the reference values that are required to make an adequate comparison, the PDR for a V2V VLC link has been assessed, in the absence of any interfering nodes. The simulations are made assuming a seven-lane straight road, with the *R_X_* located in the middle lane (lane 4), whereas the intended *T_X_* is placed along all the scenarios lane by lane. The acquired findings are shown in Figure 4, where the horizontal and vertical axes denote the longitudinal (i.e., distance from the receiver) and lateral (i.e., lane number) locations of *T_X_*, respectively. Therefore, Figure 4 illustrates the achieved PDR result for each *T_X_* location. As expected, the longest communication range is achieved when the transmitter and receiver are located on the same lane (i.e., the lateral distance is 0 m). In such conditions, a PDR higher than 90% is attained up to 32 m of longitudinal distance. In the case that the transmitter is located on the adjacent lanes to the right or left, more than 90 percent of PDR can be achieved between 2 and 31 m of longitudinal distance. Next, when the transmitter is found on a second adjacent lane, a PDR higher than 90 percent can be achieved within the 5 to 30 m longitudinal distances. Lastly, a PDR above the 90 percent target is achieved for 8 to 27 m when the transmitter is on the third adjacent lane. These results reflect the limitations imposed by the mandatory LoS condition associated with VLC, and also indicate a rather wide area where the PDR can be maintained within the platooning requirements limits.

### 2.3. Performance Modeling in Multi-User Interference Conditions

**Figure 5:**
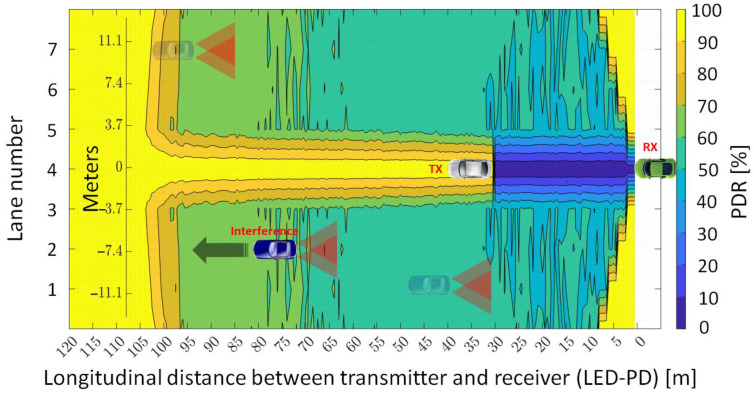
Simulation results showing the packet delivery ratio mapping for a 7-lane road in neighboring V2V VLC interference conditions.

**Figure 6:**
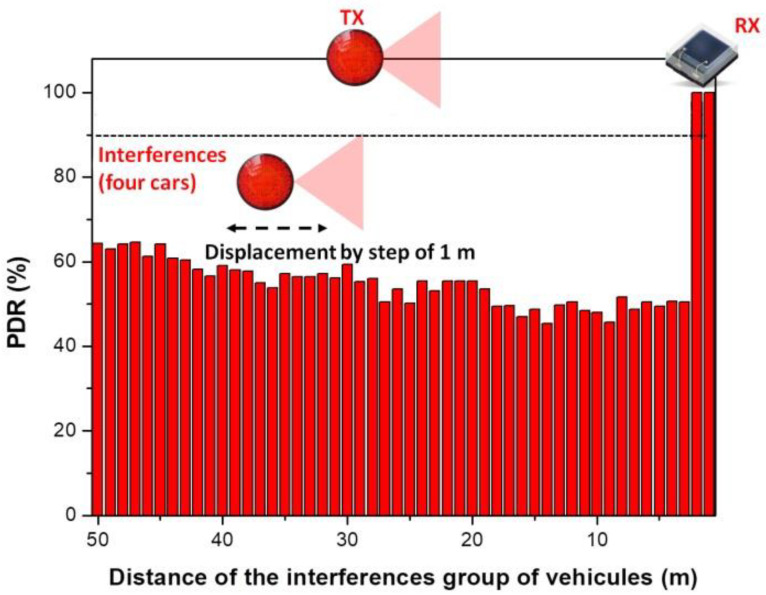
Simulation results showing the packet delivery ratio evolution in a four interference vehicles scenario, for the case when the intended VLC transmitter–VLC receiver distance is of 30 m.

Now that the reference PDR distribution with no neighboring V2V links interference has been determined, the evaluation process continues with the inclusion of the interference vehicle. Therefore, the simulation is run in order to obtain the PDR map in multi-user interference conditions. For these simulations, the transmitter and receiver vehicles are placed in the center lane (lane 4) with an inter-vehicle distance of 30 m, so that the *T_X_* is at the limit of the communication zone and the interference zone is, therefore, evaluated in the worst case. The interference transmitter changes its position with respect to the intended receiver in steps of 0.5 m, within all the scenarios, lane by lane. The results of the simulation are available in Figure 5 and illustrate the high impact an interfering vehicle has on the V2V link. As one can see, simulation results indicate that in the absence of any preventive measures, PDR below 90% are encountered for almost the entire service area. Moreover, if one considers that in the real-life scenario the link will be facing more than one interference vehicle, the expected PDR could be even worse. Therefore, in order to evaluate this scenario as well, the number of interference vehicles has been increased from one to four. In this case, the intended *T_X_* − *R_X_* VLC link was relocated to lane 1, the four interference vehicles were located on lanes two to five, whereas the rest of the simulation parameters remained unchanged (see Table 1). For these simulations, the four interference vehicles are placed at the same longitudinal distance with respect to the intended VLC receiver. Next, this distance is gradually increased from 1 to 50 m, in 1 m steps. The simulation results are represented in Figure 6 and show that the interference effect generated by the four vehicles is not significantly worse compared to the case when only one interference vehicle has been considered. Thus, in this case, the PDR drops at values around 50% when the four interference vehicles are 3 to 18 m away from the intended VLC receiver, fluctuates between 50–55% for distances between 19 and 37 m, and increases at no more than 57–64% in the 38–50 m distance interval. Therefore, as one can see, although the effect of four interference vehicles is not significantly worse compared to the effect of only one vehicle, the PDR is maintained below the 90% target even after the interference vehicles got further away at 50 m.

**Figure 7:**
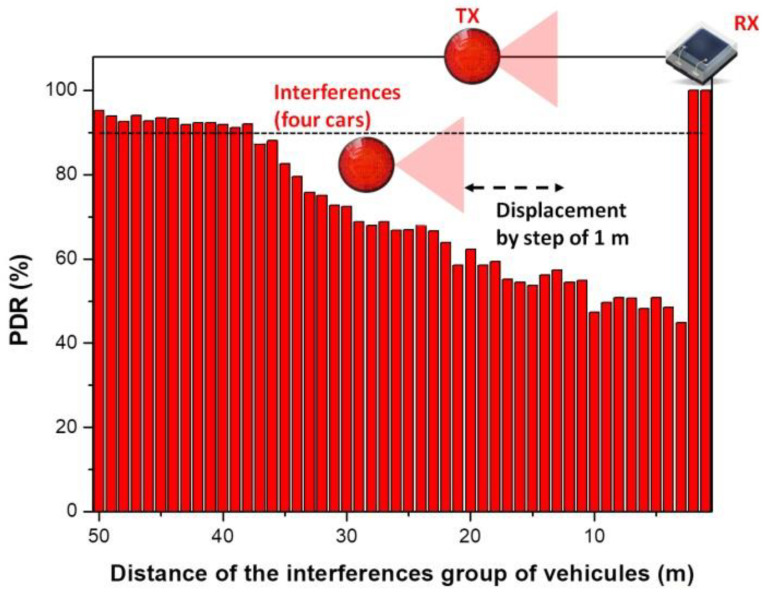
Simulation results showing the packet delivery ratio evolution in a four interference vehicles scenario, for the case when the intended VLC transmitter–VLC receiver distance is decreased at 20 m.

As previously mentioned, the 30 m *T_X_* − *R_X_* VLC distance was considered as this distance is close to the communication limit considered for this scenario, as shown in Section 2.2. Next, in order to evaluate the effect of the four interference vehicles on an improved SINR link, the *T_X_* − *R_X_* VLC distance was reduced from 30 to 20 m, and the simulation process has been started again. The results for this case are illustrated in Figure 7 and they show that when the four interference vehicles are no more than 10 m away, the effect on the PDR ratio is still rather low, at 50% values. Nevertheless, as the interference vehicles are getting further, the PDR is gradually improving, re-entering in the 90% target after the interference vehicles get more than 37 m away.

On the other hand, vehicles can only be located in distinct lanes when considering the V2V VLC link on a multi-lane straight road. Therefore, the lengths of each lane in the MUI are determined (see Figure 3). Since *d_ir_* represents the longitudinal distance from the receiver to the MUI zone limit (see Equation (8)), it can be assumed that *d_ir_* ≫ *L_W_*, where, *L_W_* is the lane width (see Figure 3), where *d_Lx_* are the lengths of individual lanes in the MUI area which are given by Equation (9).

(9) $$d_{L_{x}}=d_{ir}-j\times L_{W}\times \cot\left(\psi \right),0\leq j\leq L_{x},$$

where *L_x_* represents the number of lanes on the receiver’s right or left side, and *cot*(*ψ*) is the cotangent of the *ψ* angle. The total length of lanes inside the MUI area is then determined based on Equation (10).

(10) $$L_{tot}=d_{ir}+\sum_{L_{x}=1}^{L_{x_{1}}}d_{L_{x_{1}}}+\sum_{L_{x}=1}^{L_{x_{r}}}d_{L_{x_{r}},}$$

Here, *L_xl_* (resp. *L_xr_*) represents the number of adjacent lanes on the receiver’s left (resp. right) side. Therefore, the probability of receiving interfering signals on the lanes in the MUI area by addressing an intended transmitter and a receiver at *d_tr_* distance with the LOS condition can now be determined. For this aim, automobiles on a highway are considered to follow a Poisson distribution having a density *β* (vehicles/meters/lane), which is commonly considered for highway traffic [39], whereas the possibility of having *i* vehicles on an l-kilometer stretch road is given by Equation (11) [40].

(11) $$P\left(i,l\right)=\frac{{\left(\beta l\right)}^{i}e^{-\beta l}}{i!},$$

The channel access probability of each vehicle is symbolized by *τ*, and it is established by supposing that vehicles produce messages on a regular basis and using *T_tx_* as the average packet transmission time. Consequently, *τ* can be determined based on Equation (12).

(12) $$\tau =\frac{T_{tx}}{T_{interval},}$$

**Figure 8:**
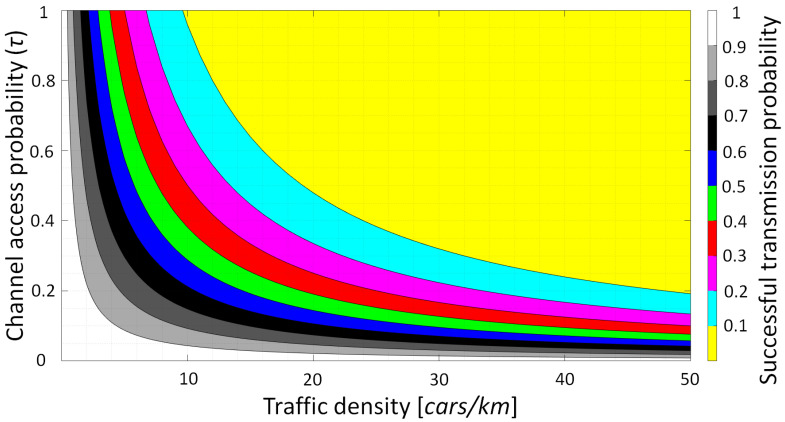
Representation of the successful communication probability on a 7-lane road.

Here, *T_interval_* is the message-generating time interval. The results shown in Figure 8 indicate that when there is no MUI control, i.e., no MAC, adequate PDR performance can only be accomplished at very low traffic density and low message-generation rates. More precisely, to achieve 90% PDR at a message generation rate of 0.8, the traffic density for a 7-lane scenario must be lower than two vehicles/Km. Then, to maintain the PDR higher than 90% in a 50 vehicles/Km scenario, the message generation rate *τ* must be lower than 0.01. Even though these results are rather pessimistic, they do emphasize the necessity for a MAC protocol for VLC, and that additional work is required in order to have reliable V2V VLC links.

## 3. Discussion on the Vehicular Visible Light Communication Prototype Used for the Experimental Evaluation

### 3.1. Debate on the Visible Light Communications Prototype Used for the Experimental Evaluation

**Figure 9:**
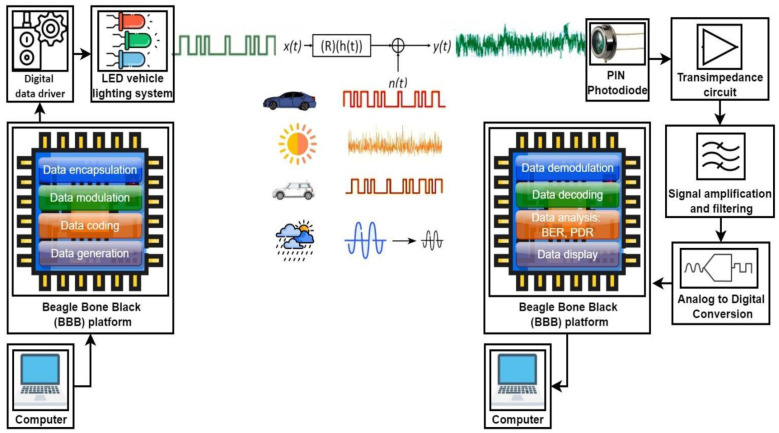
Schematic of the vehicular VLC prototype used in the experimental evaluation process.

As revealed in Section 2, neighboring V2V VLC links can influence each other, affecting the PDR performances. After establishing this fact based on analytical and simulation means, the next sections aim to set the basis for an experimental evaluation of this phenomenon. Therefore, in order to determine the effect of neighboring vehicular VLC transmitters on the performance of nearby VLC links, a hardware VLC prototype has been implemented. Figure 9 presents the block diagram of the proposed prototype, showing the VLC transmitter and the VLC receiver components, while also emphasizing the impact of the vehicular VLC channel.

For simplicity and time-efficiency reasons, the VLC prototype has been developed on a Beagle Bone Black (BBB) platform. The BBB is a low-cost, community-supported development platform for developers [41]. This board is able to manage all digital tasks such as networking, coding, and decoding the information, benefiting from the Linux operating system facilities. The BBB platform is interconnected with a computer, and together they construct the digital information to be sent. Thus, the BBB platform transforms the data to send into a binary string and performs the coding and the modulation of the data, in accordance with the specified requirements. The software solution is developed as a Linux driver that directly communicates with the cape and the Linux networking stack. The VLC interface is configured in OpenVLC as a new communication interface that can benefit from a wide range of Linux services. The driver makes use of the BBB platform’s Programmable Real-time Units (PRU) [42]. Next, the output signal is processed by a driver which converts the digital signals generated by the BBB platform into beams of the modulated LED light.

**Figure 10:**
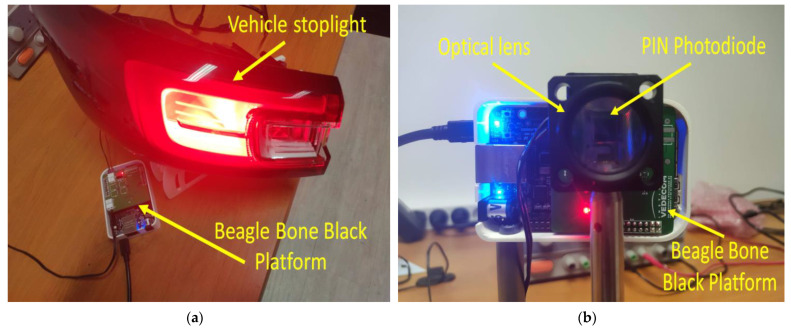
Hardware implementation of the vehicular visible light communications prototype: (**a**) vehicle stoplight VLC transmitter; (**b**) VLC receiver.

At the receiver side, the light beam reaches the photosensitive element—a reversed bias PIN photodiode connected to a transimpedance circuit which transforms the incident light into an electrical current, and then into a signal that will be further processed in order to extract the data. In a simple form, signal processing refers to signal amplification from mV levels to a few volt levels and signal band-pass filtering that removes the DC component, the low-frequency noise components introduced by incandescent and fluorescent light sources, and also high-frequency noise components such as light-generated shot noise and electronic component-generated thermal noise. For the best results, the band-pass region is established in accordance with the selected code’s power spectral distribution for the given data rate. Next, the signal is digitized and fed to the BBB platform which extracts and processes the data. It should be emphasized here that the challenges associated with the development of the analog front end for vehicular VLC applications are related to the correct tuning of parameters of the transimpedance amplifier gain with respect to the parasitic capacitance of the circuit, as the properties of the selected electronic components (photodiode, operational amplifier, etc.) such as internal capacitance, wavelength response, FOV, bandwidth. The hardware implementation of the VLC prototype is shown in Figure 10, illustrating the vehicle stop light VLC transmitter and the VLC receiver. In addition to the vehicle stop light VLC transmitter, another VLC transmitter developed based on a vehicle headlight has also been implemented.

In the end of this section, it should be emphasized that as this article is not focused on proposing a new vehicular VLC hardware design, these aspects have not been addressed in detail. Nevertheless, additional information and recommendations that are useful in vehicular VLC systems design and implementation can be found in [14,15,16,17,18,19,20,21,22].

### 3.2. Evaluating the Vehicular VLC Link in Friendly Conditions

**Figure 11:**
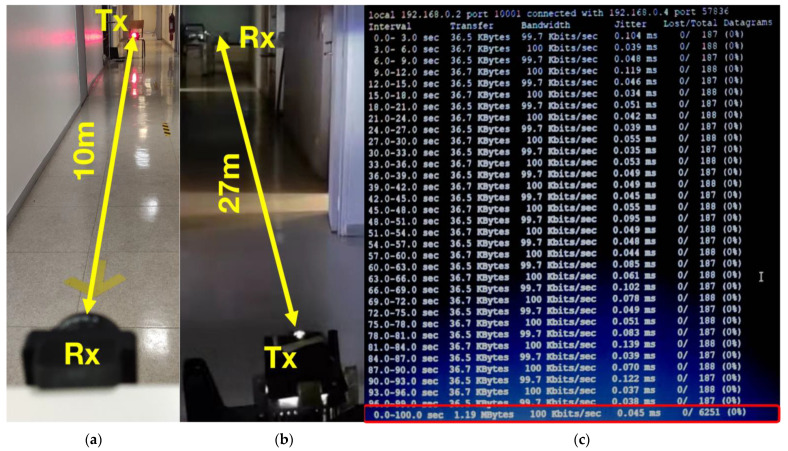
Experimental setup showing the VLC system communication range and communication parameters: (**a**) setup with a vehicle tail-light VLC transmitter enabling a 10 m communication range; (**b**) setup with a headlamp transmitter enabling a 27 m communication range; (**c**) screenshot of iperf tool showing a 100 kbps data rate and no lost datagrams.

Before moving to the experimental evaluation of the mutual interference effects on vehicular VLC performances, the VLC prototype has been evaluated in friendly conditions (Figure 11). This intermediate evaluation process is required in order to provide the reference for the next experimental evaluation section. As previously mentioned, the BBB board and the associated BBB platform enable a simple and fast configuration of the VLC link through Secure Socket Shell (SSH) service and the associated OS. The BBB platform also provides communication parameters analysis, such as information concerning the throughput and the lost packets over a certain transmission period. These functions are enabled when the iperf utility is run in client mode at the *T_X_* end and in server mode at the *R_X_* terminal with the User Datagram Protocol (UDP) setup.

For all the tests, 36.6 kb datagrams have been transmitted at a 100 kb/s data rate (Figure 11c). In this configuration, the maximum communication distance was limited to 10 m (Figure 11a) when using the stoplight VLC as the transmitter and to 15 m when using the headlamp as the VLC transmitter. In the next step, a Fresnel lens has been added at the VLC receiver level. The Fresnel lens captures a larger amount of light which is focused on the VLC receiver photoelement’s surface and thus enables a longer communication range. Thus, the larger light surface collecting area enables the system to increase the communication distance from 10 to 13 m in the case of the stoplight VLC transmitter and from 15 to 27 m in the case of the headlamp VLC transmitter (Figure 11b). Nevertheless, it should be mentioned here that the Fresnel lens reduced the VLC receiver FOV, affecting the system’s ability to work in the mobile vehicular VLC scenario. Therefore, the narrow FOV only enables a V2V link for the case when the vehicles are located in the same lane and relatively aligned. However, as the two vehicles are moving and the alignment is lost, the communication link is no longer sustained. This points out that a narrow FOV can be a solution to prevent interference from other vehicles (i.e., VLC transmitters) located on different lanes. However, such an approach becomes mostly unsuitable as it affects the compatibility with vehicular VLC applications.

It should be mentioned here that a significantly longer communications distance can be achieved by vehicular VLC systems as in the case of [22], where a 75 meters’ communication range has been achieved for a standard vehicle stoplight VLC transmitter, or in the case of [16], where a 185 meters’ communication distance has been achieved for a standard vehicle headlamp VLC transmitter. For such results, the complexity of the VLC prototype should be increased. Nevertheless, as this article is focused on interference investigation and not on demonstrating record communication ranges, these results can be considered satisfactory for the purpose of this aim.

### 3.3. Evaluating the Vehicular VLC Link in Interference Conditions

**Figure 12:**
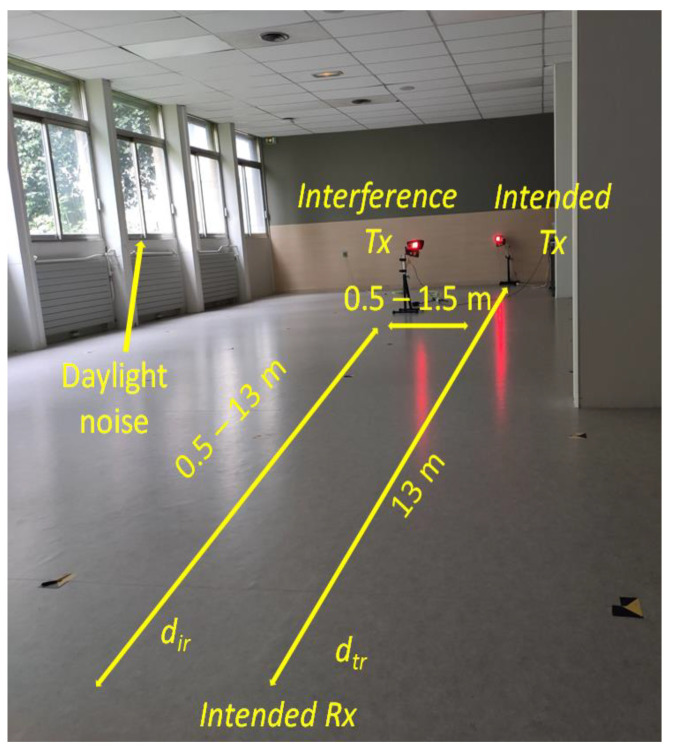
Experimental multi-user interference testing setup: VLC transmitter and VLC receiver are aligned and maintained at constant distance, whereas the interference VLC transmitter position with respect to the V2V link changes in both longitudinal and lateral distances.

**Figure 13:**
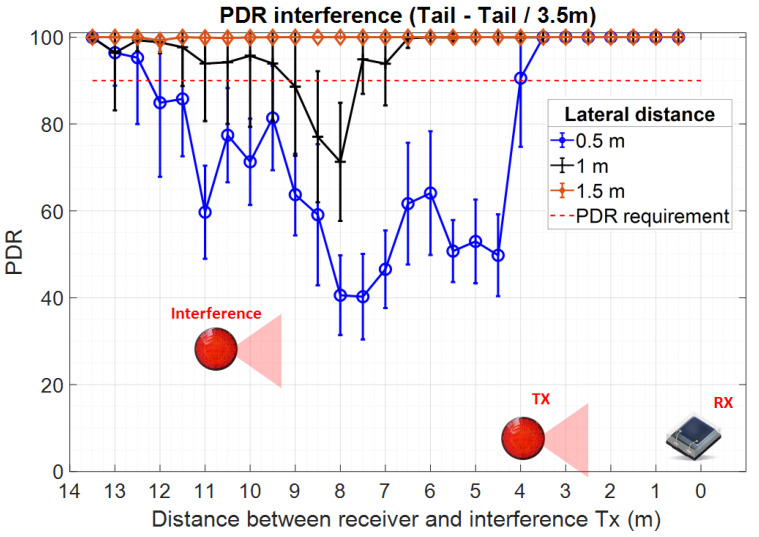
Experimental results showing the interference effect when the intended transmitter tail-light is located at 3.5 m with respect to the intended receiver, whereas the interference transmitter (tail-light) located at a lateral distance of 0.5, 1, and 1.5 m, moves longitudinally from 0 to 13 m.

**Figure 14:**
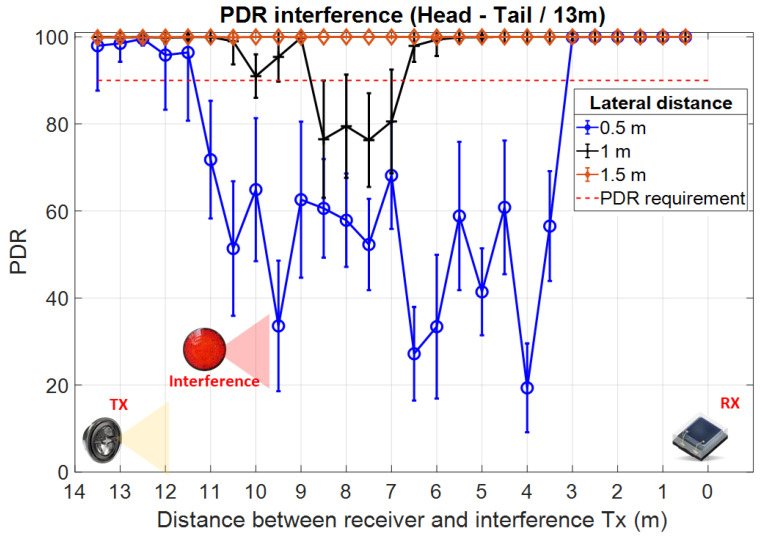
Experimental results showing the interference effect when intended headlamp transmitter is located at 13 m from the intended receiver, whereas the interference transmitter (tail-light) located at lateral distances of 0.5, 1, and 1.5 m, moves longitudinally from 0 to 13 m.

Once the reference data became available, the following section presents the experimental results investigating the effect of multiple vehicular VLC transmitters on the overall VLC link parameters. These tests have been performed in laboratory conditions in a 20 × 10 m space. Different from the previous reference tests, these experiments were performed in the presence of moderate daylight (i.e., 1500–2000 lux) coming through the windows. Figure 12 illustrates the experimental testing setup used for these measurements. Due to the lack of space and platooning consideration application requirements, these tests have been performed for a V2V communications range of only 3.5 m. The short communication range can be considered as a specific use case in which the useful signal has a relatively high SNR level prior to other optical interference sources being added. Within this setup, a second VLC transmitter has been added as an interference transmitter. Therefore, the position of the intended transmitter and receiver remained static (i.e., 3.5 m V2V distance), whereas the position of the interference transmitter has been gradually adjusted by steps of 0.5 m, over three different lateral distances, 0.5, 1, and 1.5 m. This setup is equivalent to the case when vehicles are traveling in a platoon at short inter-vehicle distances and a vehicle on a neighboring lane overtakes the platoon, or the platoon overtakes other vehicles. The experimental results showing the PDR for this experimental setup are shown in Figure 13. Next, in order to evaluate the effect of the interfering transmitter in longer V2V links, the stoplight VLC transmitter has been replaced by the headlight VLC transmitter and the V2V range has been increased to 13 m. Similar to previous tests, the interference transmitter was a tail-light located to the right of the intended transmitter. The experimental results for these experiments are summarized in Figure 14.

Similar to the simulation results, the experimental results confirm that without any action, neighboring VLC transmitters can have a high impact on the communication link performances, significantly affecting the PDR. These results also showed that this impact is highly dependent on the amount of interference that reaches the VLC receiver level, which is influenced by the longitudinal and the lateral distances between the interfering vehicle and intended receiver. As shown in Figure 13 and in Figure 14, this effect is very intense even when the intended transmitter is closer to the VLC receiver than the interference transmitter. The experimental results have also shown that part of the interference can be suppressed with the help of the optical configuration. These tests have shown that with a Fresnel lens of 25 mm diameter and a focal point of 10 mm the communication range increases by a few meters and the interference coming from a lateral displacement of 1.5 m disappear.

### 3.4. Debate on the Experimental Results and Article Findings

This section aimed at experimentally investigating the effects of multi-user interference on the V2V VLC link PDR. The experimental testing conditions aimed to follow the line of a real platooning scenario. Thus, the effect of interference was tested for two relevant scenarios. In the first one, the V2V distance is quite short (i.e., 3.5 m), and therefore, the interference VLC transmitter is located at a longer distance from the receiver, compared to the position of the intended VLC transmitter. In the second case, the V2V distance is increased to 13 m (i.e., compared to 3.5 m initially), and so, the effect of the interference transmitter is investigated for the case when this one is located closer to the intended receiver compared to the intended transmitter. In both situations, the effect of lateral distance has been also investigated, considering 0.5, 1, and 1.5 m lateral distances. Thus, it can be considered that the experimental testing scenario is widely covering the main situations which could influence multi-user interference.

The experimental results confirm several interesting points. First, one can see that for both experimental setups, interference do not affect link performances when the interference TX—receiver RX distance is of only a few meters. This can be attributed to the fact that in such cases, the limited VLC receiver FOV eliminates the TX signal to reach the VLC receiver photosensitive element. Next, as the distance increases, and the TX interference transmitter comes within the VLC receiver LoS, the PDR suddenly drops. As expected, the experimental results confirm that in such a case, the PDR is less affected when the V2V distance is shorter and thus, the SINR level is higher. This explains why the impact of the interference vehicle is so high in the second case (i.e., V2V distance of 13 m—Figure 14). As one can see, in these conditions, the PDR drops from values close to 100% to values around 20%, compared to a 50% PDR in the first case (i.e., V2V distance of 3.5 m—Figure 13). Following, as the distance between the TX interference transmitter and the RX receiver is increasing, the low PDR is initially maintained as the SINR level is maintained. In this case, the distance increase is compensated by the incidence angle decrease, resulting in a relatively similar SINR. Then, as the TX interference transmitter and the RX receiver distance continues to increase, the SINR increases and the PDR level gradually increases as well, re-entering in the targeted 90% limit. Therefore, these results confirm the simulation results illustrated in Figure 2 which link the PDR performances to the SINR level. Similar to simulation results, the experimental evaluation has shown that under intense interference exposure, the PDR decreases below the imposed 90% limit for almost the entire area. Exceptions to this rule are the cases when the interference are generated by vehicles located outside the VLC receiver FOV (i.e., at short distances and angles outside the VLC receiver FOV). On this topic, the experimental results also showed that the effect of interference gradually decreases as the lateral distance is increasing which is given by the fact that the optical power reaching the VLC receiver has an angular cosine dependency. This effect is highlighted when comparing the results of the 0.5 and 1 m lateral distance experiments. Nevertheless, in the case of the current experimental determinations, the high PDR observed for the 1.5 m lateral distance setup is mostly the result of relatively high directivity associated with the VLC interference transmitter. On the other hand, existing regulations do not provide clear standards for the vehicle stoplight systems. Therefore, as shown in [22,43,44], the radiation pattern of existing vehicle stoplight systems can vary considerably. Additionally, the high PDR at 1.5 m lateral distance is the result of a relatively narrow FOV VLC receiver. Therefore, the experimental results have shown that from a PHY layer perspective, the effect of interference can be partially mitigated with the help of a narrow FOV. On the other hand, as this approach limits the mobility of the system, a context-adaptive approach based on an adjustable FOV receiver could be used, as suggested in [45,46]. In such a case, the VLC receiver could adapt the FOV in accordance with different factors, maximizing its performance.

### 3.5. Multi-User Interference Mitigation Techniques in Vehicular Visible Light Communications Networks

This article has provided a complex analysis demonstrating the disruptive effects of MUI on the performance of vehicular VLC systems. Nevertheless, even if these issues exist, it should be also mentioned that the research community is working on identifying solutions to mitigate the effects. As summarized in [32,47], these solutions are focusing the development of multiple user access techniques and are mainly based on Frequency Division Multiplexing (FDM) including here the Orthogonal FDM approach, Time Division Multiplexing (TDM), Code Division Multiplexing (CDM), and Space Division Multiplexing (SDM). At this point, Orthogonal Frequency Division Multiple Access (OFDMA) seem to be the most advanced solution in this domain [48], being somehow affected by a rather complex implementation. Code Division Multiple Access (CDMA) is also a popular solution which benefits from a simpler implementation and relatively decent performance. On the other hand, it must be emphasized that the work focused on multi-user access and on multi-user interference mitigation is mostly orientated toward indoor VLC applications rather than vehicular VLC utilization. Examples of studies focused on vehicular VLC applications are found in [30,31], where an optical CDMA solution is proposed, or in [29,49], where solutions based on Space-Division Multiple Access (SDMA) using vehicle matrix headlights are analyzed. Additionally, [33] provides a detailed analysis focused on the problems associated with MUI in vehicular VLC applications and on the benefits of optical CDMA utilization.

## 4. Conclusions

In the context in which vehicular VLC has significantly evolved in terms of physical layer performances, and have overcome some of the previously defined challenges, new trials emerge. Thus, this article provided an intensive evaluation concerning the disruptive effect generated by the neighboring V2V VLC links. The simulation results have shown that the PDR performances are strongly linked with the SINR, and that in multi-user interference conditions, the PDR is significantly affected. Thus, PDR results below the 90% target are provided within the entire service area. This means that without preventing action, V2V VLC links are highly vulnerable to multi-user interference. These simulation results are also supported by the experimental investigations which also demonstrated the increased vulnerability to interference from neighboring V2V VLC links. Therefore, simulations and experimental results indicate the importance of a MAC protocol that manages the interference, minimizing data packet losses and improving the link resilience in autonomous vehicle platoons.

As far as we know, this article is the first intensive work that provides intensive simulation and experimental results that demonstrate the severe perturbing effect generated by multi-user V2V VLC links. Future work on this topic will focus on the implementation of MAC protocols and their experimental testing.

## Figures and Tables

**Table 1:** MATLAB simulation parameters.

Parameter	Value
PD reference	SFH-206 k
*A_r_*	7.02 mm^2^
PD efficiency	0.62 A/W
VLC receiver FOV (*ψ_e_*)	60°
LED half angle (*α*)	20°
LED-PD lateral distance (*d_L_*)	3 m
Vehicle width (*d_w_*)	2 m
Vehicle length (*d_len_*)	4.5 m
PD capacitance	72 pF/m^2^
Transmission frequency	500 kHz
Transmission power	2 Watt (vehicle tail-light)
Packet size (*L_b_*)	100 bits
Lane width (*L_W_*)	3.7 m

## Data Availability

Not applicable.
